# Upconversion time-stretch infrared spectroscopy

**DOI:** 10.1038/s41377-023-01096-4

**Published:** 2023-03-04

**Authors:** Kazuki Hashimoto, Takuma Nakamura, Takahiro Kageyama, Venkata Ramaiah Badarla, Hiroyuki Shimada, Ryoich Horisaki, Takuro Ideguchi

**Affiliations:** 1 Institute for Photon Science and Technology, The University of Tokyo, Tokyo, 113-0033 Japan; 2 Department of Physics, The University of Tokyo, Tokyo, 113-0033 Japan; 3 Graduate School of Information Science and Technology, The University of Tokyo, Tokyo, 113-8656 Japan

**Keywords:** Infrared spectroscopy, Ultrafast photonics

## Abstract

High-speed measurement confronts the extreme speed limit when the signal becomes comparable to the noise level. In the context of broadband mid-infrared spectroscopy, state-of-the-art ultrafast Fourier-transform infrared spectrometers, in particular dual-comb spectrometers, have improved the measurement rate up to a few MSpectra s^−1^, which is limited by the signal-to-noise ratio. Time-stretch infrared spectroscopy, an emerging ultrafast frequency-swept mid-infrared spectroscopy technique, has shown a record-high rate of 80 MSpectra s^−1^ with an intrinsically higher signal-to-noise ratio than Fourier-transform spectroscopy by more than the square-root of the number of spectral elements. However, it can measure no more than ~30 spectral elements with a low resolution of several cm^−1^. Here, we significantly increase the measurable number of spectral elements to more than 1000 by incorporating a nonlinear upconversion process. The one-to-one mapping of a broadband spectrum from the mid-infrared to the near-infrared telecommunication region enables low-loss time-stretching with a single-mode optical fiber and low-noise signal detection with a high-bandwidth photoreceiver. We demonstrate high-resolution mid-infrared spectroscopy of gas-phase methane molecules with a high resolution of 0.017 cm^−1^. This unprecedentedly high-speed vibrational spectroscopy technique would satisfy various unmet needs in experimental molecular science, e.g., measuring ultrafast dynamics of irreversible phenomena, statistically analyzing a large amount of heterogeneous spectral data, or taking broadband hyperspectral images at a high frame rate.

## Abstract (web summary)

We develop upconversion time-stretch infrared spectroscopy, which enables high-speed and high-resolution broadband mid-infrared spectroscopy with over 1000 spectral elements at a rate of more than 10 MSpectra s^−1^.

## Graphical abstract

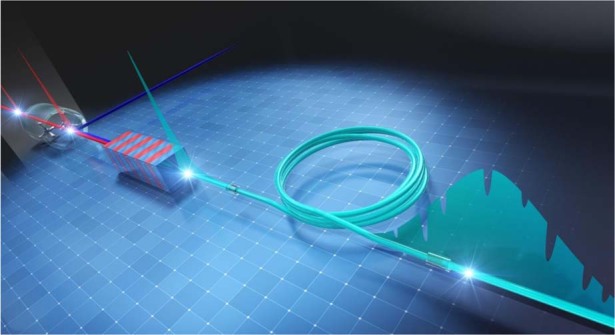

## Introduction

Broadband mid-infrared (MIR) spectroscopy^1^ is a powerful non-invasive tool for identifying molecular species and sensing subtle changes in molecular structures that reflect environmental conditions, and various applications have been widely investigated, such as environmental gas monitoring^2–4^, combustion analysis^5,6^, photoreactive protein analysis^7^, liquid biopsy^8,9^, breath diagnosis^10,11^, etc. One of the promising directions of instrumental development of broadband MIR spectroscopy is increasing the measurement speed because normal vibration modes of molecules have huge MIR absorption cross-sections, which are orders of magnitude larger than Raman scattering cross-sections. One approach for that is parallel signal detection using a sensor array with a grating-based dispersive spectrometer, but the spectral measurement rate is limited by the readout rate of the sensor, which is typically up to ~1 kSpectra s^−1^ ^12,13^. Another approach is increasing the scan rate of a Fourier-transform spectrometer by taking advantage of the high bandwidth of a photodetector. Advanced Fourier-transform infrared spectroscopy (FTIR) such as MIR dual-comb spectroscopy (MIR-DCS)^3–7,14–22^, rapid-scan FTIR^23^, and phase-controlled FTIR^24^ has remarkably improved the measurement scan rate up to a few MSpectra s^−1^ ^7,18,20^. These high-speed FTIR techniques could open doors for applications such as measuring non-repetitive rapid phenomena at high temporal resolution and making statistical analyses of a significantly large amount of spectral data. However, the measurement rate has already hit the theoretical maximum limited by the signal-to-noise ratio (SNR). Therefore, to improve the measurement rate further, one needs a method with a fundamentally higher SNR.

Frequency-swept spectroscopy (FSS)^25–27^ is a method having higher SNR than FTIR, where a broadband spectrum is measured by sweeping the laser frequency. It has more than ~$\sqrt M$ (*M*: number of spectral elements) times higher SNR than FTIR^28^ due to the less noise per spectral element (theoretical description is shown in Supplementary Note 8). Therefore, FSS techniques have the potential to increase the spectral measurement rate, but, to the best of our knowledge, the highest scan rate of MIR-FSS with a frequency-swept MIR laser was 250 kSpectra s^−1^ ^26^, which can measure two spectral elements only. We proposed that a time-stretched pulse could be used as a high-speed frequency-swept laser and demonstrated time-stretch infrared spectroscopy (TSIR) at the record spectral measurement rate of 80 MSpectra s^−1^ ^29^. Although the developed system significantly improved the measurement rate, the measurable number of spectral elements was limited to about 30 (the spectral resolution was 7.7 cm^−1^), mainly due to the large loss in time-stretching with a free-space angular-chirp-enhanced delay line (FACED)^30^ and the low sensitivity in MIR photodetection. With the small number of spectral elements, one does not gain the full advantage of broadband MIR spectroscopy, i.e., the high SNR multiplex spectral measurement with a large number of spectral elements. Furthermore, the low spectral resolution does not allow one to apply it to gas-phase spectroscopy.

In this work, we develop upconversion TSIR (UC-TSIR) and demonstrate high-speed and high-resolution broadband MIR spectroscopy with spectral elements of more than 1000 at a rate of above 10 MSpectra s^−1^. The nonlinear upconversion^31–34^ via difference frequency generation (DFG) allows the implementation of time-stretching and photodetection in the near-infrared (NIR) telecommunication region, where high-quality optics and optical devices are well developed. It provides superior advantages for TSIR: (1) low-loss and large pulse-stretching with a telecommunication-grade optical fiber^35,36^ and (2) low-noise and high-bandwidth pulse detection with an InGaAs photodetector, enabling high-speed, high-resolution, and high-content MIR spectroscopy. As a proof of concept demonstration, we measure gaseous CH_4_ molecules with different pulse-stretching conditions. For a demonstration of high-speed capability, it is operated at 80 MSpectra s^−1^ with a spectral resolution of 0.10 cm^−1^ and −10-dB bandwidth of 20 cm^−1^ (200 spectral elements). The threshold level of spectral bandwidth (−10 dB in this case) is determined where the single-shot SNR becomes 1. For a demonstration of high spectral resolution, it is operated at a rate of 10 MSpectra s^−1^ with spectral resolutions and bandwidths of 0.034 and 26 cm^−1^ at −10 dB level (760 spectral elements), and 0.017 and 17 cm^−1^ at −8 dB level (1000 spectral elements).

## Results

**Fig. 1:**
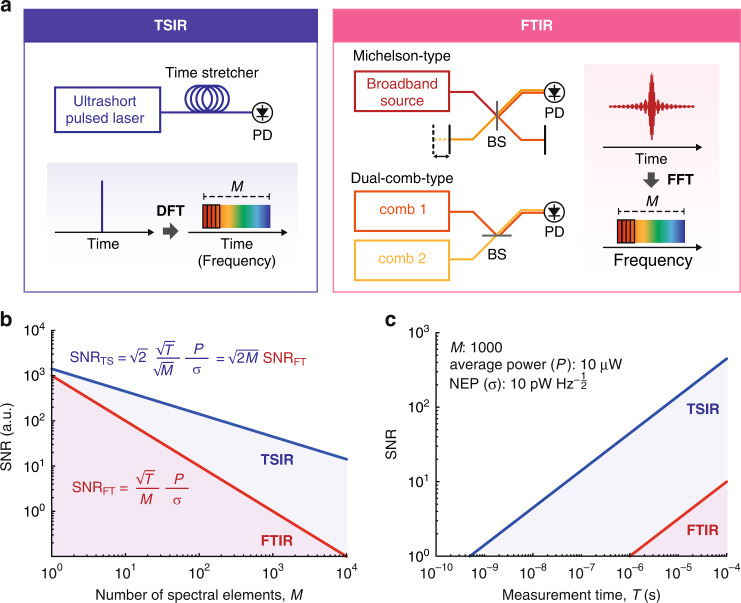
Comparison of working principle and SNR between TSIR and FTIR. **a** Working principle of TSIR and FTIR. DFT dispersive Fourier-transform, FFT Fast-Fourier transform, BS beamsplitter, PD photodetector. **b** SNR dependence on the number of spectral elements, *M* (Blue: TSIR, Red: FTIR). *P*: average detection power, *σ* system’s overall noise equivalent power. **c** SNR dependence on measurement time, *T* (Blue: TSIR, Red: FTIR). The SNR is calculated for *M* = 1000 with typical values of *P* = 10 µW and *σ* =10 ${{{\mathrm{pW}}}}$ ${{{\mathrm{Hz}}}}^{ - \frac{1}{2}}$. NEP noise-equivalent power

Figure 1 illustrates a comparison of the working principle and SNR between TSIR and FTIR. A TSIR spectrometer measures spectra directly in the time domain by photonic time-stretch, also known as dispersive Fourier-transformation (DFT), while an FTIR spectrometer measures temporal interferograms and converts them to spectra by fast Fourier-transformation (FFT). In TSIR, the noise of a sampled data point directly becomes that of a corresponding spectral element, whereas, in FTIR, the noise of all the data points of an interferogram contributes to that of a spectral element via FFT. The difference in the amount of noise per spectral element results in the $\sqrt M$-times higher SNR of TSIR, where *M* is the number of spectral elements. In addition, TSIR allows measuring a two-averaged spectrum within a measurement time of a single FTIR spectrum because the number of FTIR spectral elements is half the number of the sampling data points due to the Nyquist theorem, which gives an additional SNR factor of $\sqrt 2$. Furthermore, under the condition where the detector’s dynamic range limits the SNR, TSIR obtains an extra SNR advantage of a factor (1 ≤ *α* ≤ 2), which is a constant value determined by noise conditions. It comes from the FTIR’s working mechanism, where the DC signal of the interferogram consumes half of the dynamic range. In total, TSIR has $\alpha \sqrt {2M}$-times higher SNR than FTIR. The detailed theoretical description is summarized in Supplementary Note 8.

Figure 1b, c show the theoretically calculated SNR of TSIR (Eq. (S12)) and FTIR (Eq. (S21)) at *α* = 1 (the lowest value of *α*) as a function of the number of spectral elements *M* and measurement time *T*, respectively. Figure 1b visualizes TSIR has $\sqrt {2M}$-times higher SNR than FTIR, and the SNR advantage becomes significant when *M* is large. For example, TSIR can have higher SNR than FTIR by 45 when *M* = 1000. Note that the vertical axis of the SNR is an arbitrary unit, which varies depending on measurement conditions, including measurement time. Figure 1c shows the SNR dependence on measurement time *T* for *M* = 1000. Here, we calculate the SNR by assuming flat-top spectra under a measurement condition with typical parameter values: the average detection power of 10 µW, and the system’s overall noise-equivalent power of 10 ${{{\mathrm{pW}}}}$ ${{{\mathrm{Hz}}}}^{ - \frac{1}{2}}$. We assume that the detection bandwidth and the sampling rate are sufficiently high for the measurement. The SNR of TSIR becomes 1 at the measurement time of 500 ps, showing the potential to achieve a measurement rate of 2 GSpectra s^−1^. On the other hand, FTIR requires 1 µs to achieve an SNR of 1, limiting the maximum measurement rate at 1 MSpectra s^−1^, which agrees well with the results of the previous works of high-speed FTIR such as MIR-DCS^7,18,20^.

**Fig. 2:**
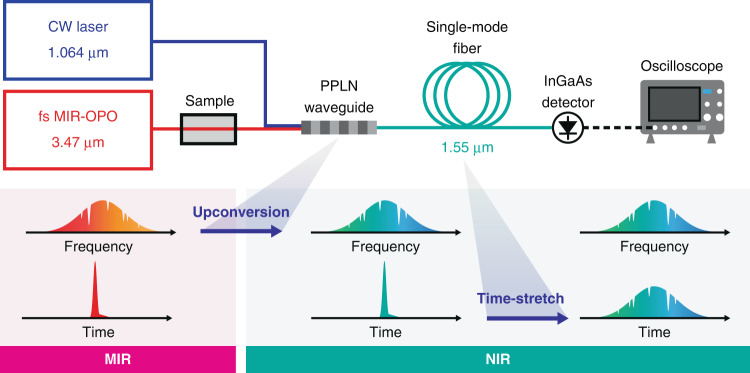
Schematic of upconversion TSIR (UC-TSIR). MIR-OPO mid-infrared optical parametric oscillator, PPLN periodically poled lithium niobate

Figure 2 illustrates a schematic of our UC-TSIR spectrometer. We use a homemade 80-MHz femtosecond MIR optical parametric oscillator (OPO) with a −10-dB spectral bandwidth of 235 cm^−1^ at a center wavelength of 3.47 µm as a broadband MIR light source. The MIR beam passing through a sample (a CH_4_ gas cell) with an average power of a few mW is spatially combined with a 1.064-µm continuous-wave (CW) laser beam with an average power of around 400 mW. The combined beams are focused onto a 20-mm-long periodically poled lithium niobate (PPLN) waveguide with an aspheric lens. The DFG process in the waveguide converts the MIR pulses to NIR pulses around 1.5 µm with an average power of 3 µW. The −10-dB spectral bandwidth of the upconverted NIR pulses is 21 cm^−1^ (5.1 nm), which is determined by the phase-matching condition in the PPLN waveguide. The use of the 1-µm CW laser for the DFG guarantees a one-to-one spectral transfer from a MIR pulse to a NIR pulse. The generated NIR pulses are coupled into a single-mode fiber with a coupling efficiency of 0.65 and optically amplified with an Er-doped fiber amplifier (EDFA). Subsequently, they are temporally stretched by dispersion-compensating fibers (DCF) with a total length of 10, 30, or 60 km. The DCF’s dispersion parameter is −0.2 ns nm^−1^ km^−1^. In large-stretching cases with a fiber length of 30 and 60 km (total dispersion of −6 and −12 ns nm^−1^, respectively), we additionally implement a pulse picker to avoid temporal overlap of adjacent pulses and a Raman amplifier to retain the signal intensity. The stretched NIR pulses pass through optical bandpass filters for spectral bandwidth adjustment. The filtered NIR pulses are detected and digitized with an 11-GHz InGaAs photodetector and a 16-GHz oscilloscope at a sampling rate of 80 GSamples s^−1^. The details of the system are described in the “Materials and methods” section and Supplementary Note 1. To avoid spectral distortions due to undesired nonlinear effects, it is essential to carefully manage the pulse energy in the PPLN waveguide and the optical fiber. The details of the nonlinear effects are described in Supplementary Notes 2 and 3.

**Fig. 3:**
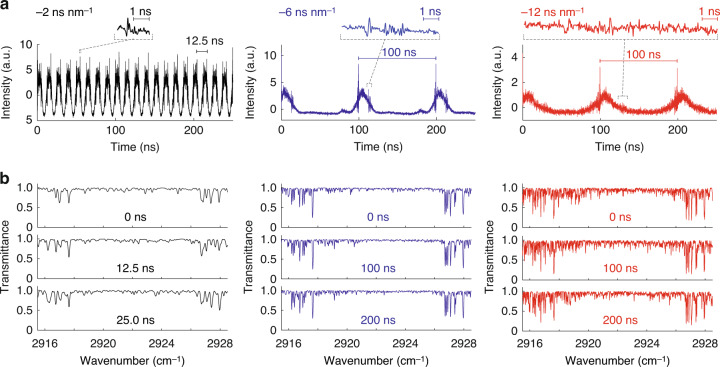
High-speed broadband MIR spectroscopy of gas-phase CH_4_ molecules with upconversion TSIR. **a** Continuously measured TSIR spectra at a rate of 80 MSpectra s^−1^ with a 10-km DCF (total dispersion of −2 ns nm^−1^) (black), 10 MSpectra s^−1^ with a 30-km DCF (total dispersion of −6 ns nm^−1^) (blue), and 10 MSpectra s^−1^ with a 60-km DCF (total dispersion of −12 ns nm^−1^) (red). The insets show enlarged views of a part of the spectra. **b** Single-shot transmittance spectra retrieved by the GD algorithm measured at 80 MSpectra s^−1^ with a 10-km DCF (black), 10 MSpectra s^−1^ with a 30-km DCF (blue), and 10 MSpectra s^−1^ with a 60-km DCF (red)

We demonstrate broadband UC-TSIR spectroscopy of gaseous CH_4_ molecules in a 50-mm-long cell with a pressure of 10 Torr. To show the high-speed capability, we first operate the TSIR spectrometer at 80 MSpectra s^−1^ by setting the fiber length to 10 km (dispersion of −2 ns nm^−1^) to stretch the spectrum over the pulses’ interval of 12.5 ns. The left panel in Fig. 3a shows temporal TSIR waveforms, that is, TSIR spectra. The TSIR spectral resolution is determined either by the pulse duration before stretching or the impulse response of the detector. In our experiments, the measured temporal width of an unstretched NIR pulse using the photodetector is 49 ps, which is determined by the impulse response of the detector (details are shown in Supplementary Note 4). Under this condition, the number of TSIR spectral elements is 200 with a −10-dB spectral bandwidth of 20 cm^−1^ (corresponding to 9 ns in the time domain) and a spectral resolution of 0.10 cm^-1^ (49 ps). Absorption lines of gaseous CH_4_ molecules appear on the spectrum, verifying the capability of high-speed and high-content broadband MIR spectroscopy. The distortions in the absorption lineshape are not due to the measurement noise but systematic interference resulting from the near-field effect of dispersive propagation in the long fiber. The near-field propagation effect can be analogically explained as a temporal version of the well-known spatial near-field diffraction pattern, known as the Fresnel diffraction pattern (see Supplementary Note 5 for details).

Next, we use longer optical fibers of 30 km (dispersion of −6 ns nm^−1^) and 60 km (dispersion of −12 ns nm^−1^) to demonstrate higher spectral resolution (the middle and right panels in Fig. 3a, respectively). The pulse repetition rate (TSIR spectral measurement rate) is set to 10 MHz with a pulse picker to avoid temporal overlaps of the stretched adjacent pulses. To keep a sufficient signal intensity throughout the long travel in the optical fiber, we implement a Raman amplifier in the stretching fiber to compensate for the fiber loss, e.g., 22 dB in a 30-km DCF. Under this condition, the number of spectral elements for 30-km DCF is 760 with a −10-dB spectral bandwidth of 26 cm^−1^ and a spectral resolution of 0.034 cm^-1^, and that for 60-km DCF is 1000 with a −8 dB spectral bandwidth of 17 cm^−1^ and a spectral resolution of 0.017 cm^−1^. We evaluate SNR by taking the standard deviation of a baseline-normalized single TSIR spectrum where large absorption lines do not exist. The baseline-normalized TSIR spectrum is calculated by dividing a measured TSIR spectrum by an envelope curve processed by Savitzky–Golay (SG) filtering^37^. The single-shot SNRs are 10 with the 30-km DCF and 6 with the 60-km DCF when the average detection powers are 21 and 12 µW, respectively. In our current system, the SNR is limited by the shot noise determined by the number of photons before the optical amplification and the amplified spontaneous emission (ASE) noise of the optical amplifiers for the average detection power above 10 µW. A detailed discussion of the SNR is described in Supplementary Note 9.

The systematic spectral distortions due to the near-field propagation can be demodulated by the iterative gradient-descent (GD) algorithm^38^. The spectral retrieval procedures are described in the “Materials and methods”. Figure 3b shows demodulated transmittance spectra of CH_4_ molecules retrieved from the TSIR spectra measured at 80 MSpectra s^−1^ with the 10-km DCF (total dispersion of −2 ns nm^−1^), 10 MSpectra s^−1^ with the 30-km DCF (total dispersion of −6 ns nm^−1^), and 10 MSpectra s^−1^ with 60-km DCF (total dispersion of −12 ns nm^−1^), respectively. The absorption lines of CH_4_ molecules are recovered with a spectral resolution of 0.12 cm^−1^ (3.6 GHz), 0.04 cm^−1^ (1.2 GHz), and 0.02 cm^−1^ (600 MHz), respectively. The wavenumber axis is downconverted from that in the NIR region, whose relative accuracy is determined by the dispersion values for pulse stretching used in the GD algorithm (see the “Materials and methods” section and Supplementary Note 7). The values of group-delay dispersion (GDD) and third-order dispersion (TOD) used in the calculation are described in “Materials and methods”. The GDD values are calibrated by comparing the measured and calculated TSIR spectra, while the TOD values are estimated from the relative dispersion slope of the DCF given in a product’s datasheet.

**Fig. 4:**
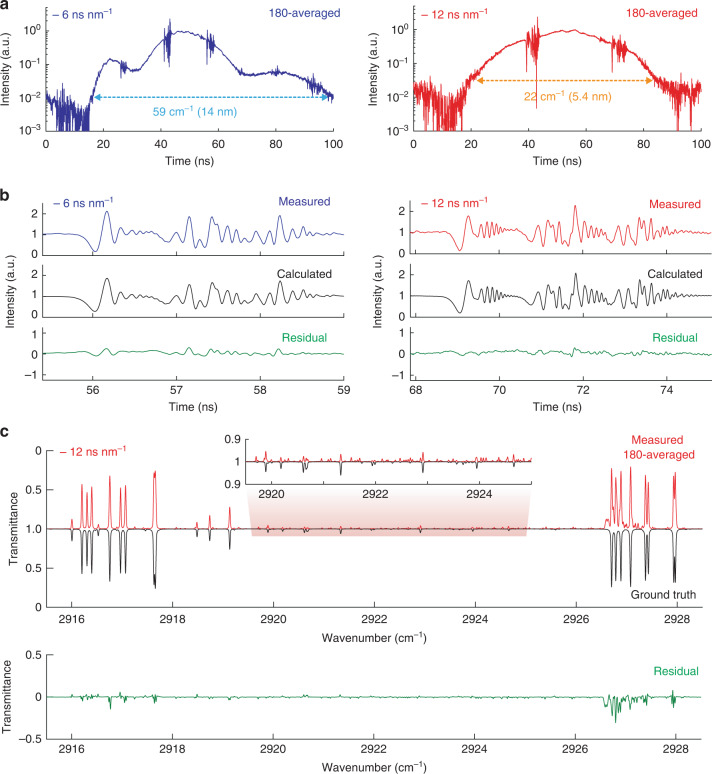
High-resolution broadband TSIR spectra of CH_4_ molecules. **a** 180-times averaged TSIR spectra measured with a 30-km DCF (dispersion of −6 ns nm^−1^) (blue) and a 60-km DCF (dispersion of −12 ns nm^−1^) (red). **b** Comparison between a part of measured and calculated TSIR spectra. The temporal baselines of the measured TSIR spectra are normalized by the envelope functions. The green plots represent the residuals. **c** A retrieved transmittance spectrum (dispersion of −12 ns nm^−1^) by the GD algorithm (red) compared to a calculated ground-truth transmittance spectrum from the HITRAN database (black). The inset shows a zoom-in view of small absorption peaks. The green plot shows residual

Figure 4a shows 180-times averaged TSIR spectra with dispersions of −6 and −12 ns nm^−1^. The pulses are stretched to 84 and 65 ns (evaluated at −20 and −15-dB intensity levels, respectively), which correspond to the spectral bandwidths of 59 cm^−1^ (14 nm) and 22 cm^−1^ (5.4 nm), respectively. We set the spectral bandwidth for each case with an optical bandpass filter. Considering the 49-ps impulse response of the photodetector, the number of TSIR spectral elements is 1700 and 1330, respectively. The averaging works well due to the high stability in the temporal axis. The standard deviation of the peak position is 9 ps, which is shorter than the oscilloscope’s sampling time resolution of 12.5 ps. It is evaluated with a spectral point at 56.16 ns of the continuously measured single-shot TSIR spectra with a 30-km DCF (details are discussed in Supplementary Note 6). Figure 4b compares the measured TSIR spectra and theoretically calculated spectra based on Eqs. (S1–S7) with parameters from the experiment and the HITRAN database^39^. The temporal baselines of the measured TSIR spectra are normalized by dividing the measured TSIR spectra by the envelope curve processed by SG filtering for a comparison with the calculated spectra. The spectral phase used in the calculation is deduced from the Kramers–Kronig (K–K) relation. The GDD and TOD values used in the calculation are 7664 ps^2^ and −48 ps^3^ for the spectrum with the dispersion of −6 ns nm^−1^, and 15,326 ps^2^ and −96 ps^3^ for the spectrum with the dispersion of −12 ns nm^−1^, respectively. The figure shows that the measured spectra agree well with the calculated ones.

Figure 4c compares the 180-averaged measured transmittance spectrum (with a 60-km DCF) retrieved with the GD algorithm and the calculated transmittance spectrum from the HITRAN database (ground truth). The absorption lines of CH_4_ molecules in the measured transmittance spectrum are in good agreement with the ground-truth spectrum at the spectral resolution of 0.02 cm^−1^ (600 MHz). As seen in the inset of the figure, several a-few-% absorption peaks are clearly observed in the retrieved spectrum. There are relatively large residuals around, e.g., 2926–2928 cm^−1^, which are likely caused by estimation error of spectral baseline because there are some bumps in the MIR baseline spectrum itself. We could suppress the residuals by using a flatter MIR spectrum without bumpy structures. Deviations of the peak positions of the absorption lines from the HITRAN are within 0.007 cm^−1^ (200 MHz) (see Supplementary Note 7 for details).

## Discussion

We make a detailed comparison between the UC-TSIR system and the previous TSIR system^29^, particularly about the performance of their time-stretchers and photodetectors. The time-stretching in UC-TSIR is made with a low-loss DCF fiber in the NIR telecommunication region, while that in the previous TSIR is made with a FACED system in the MIR region. FACED has a large loss due to the multiple reflections on flat mirrors in free space. To make a comparison, for example, if we allow 10 dB loss for the stretching, they have abilities to add dispersions of −3 ns nm^−1^ (DCF) and −20 ps nm^−1^ (FACED), respectively. Therefore, the DCF time-stretcher can stretch a pulse by two orders of magnitude longer. The fiber time-stretcher has another advantage in keeping spatial mode for a long-range (tens of km) due to the nature of waveguiding, while it is difficult to make the long-range propagation with FACED because of the beam divergence in free space. Furthermore, the fiber time-stretcher can work as an optical amplifier to compensate for the propagation loss, enabling the pulse-stretching even longer. Regarding photodetectors, UC-TSIR uses an amplified InGaAs photodetector with a responsivity of ~1 A W^−1^, while the previous TSIR system uses a quantum cascade detector (QCD) working in the MIR region with a responsivity of ~10 mA W^−1^ ^40^. The QCD’s low responsivity requires more than a few mW photodetection for capturing the signals, resulting in additional difficulty in implementing a TSIR system. With these advantages, UC-TSIR enables broadband MIR spectroscopy with a significantly large number of spectral elements and a high-spectral resolution by maintaining high-speed capability.

For a comparison to the state-of-the-art high-speed MIR-FSS, we discuss the system performances of UC-TSIR and rapid-scan external-cavity quantum cascade laser (EC-QCL) spectrometer^25–27,41^ in terms of spectral measurement rate and the measurable number of spectral elements. The fastest EC-QCL spectrometer with an acousto-optic modulator (AOM) can be operated at a rate of 1 MSpectra s^−1^ for measuring two spectral elements only^27^. The scan rate is limited by the propagation speed of acoustic waves generated by a piezoelectric transducer. The AOM-based rapid-scan EC-QCL spectrometer demonstrates MIR spectroscopy with a bandwidth of 50 cm^−1^ (two spectral elements) at a rate of 250 kHz^26^ and 200 cm^−1^ (~20 spectral elements) at 15 kHz^41^. On the other hand, UC-TSIR does not suffer from the speed limitation caused by active optical devices because of the ultrafast passive frequency sweep enabled by time-stretching. Therefore, it can measure the number of spectral elements more than 10^3^ at a measurement rate of tens of MHz, far exceeding the previous state-of-the-art.

The performance of the UC-TSIR spectrometer can be improved further with system modifications. The SNR of the current system is limited by the shot noise determined by the number of photons before the optical amplification and the ASE noise from the optical amplifier. It can be improved by several times (Supplementary Note 9) by increasing the number of upconverted photons before the optical amplification, which improves the shot-noise-limited SNR determined by the number of photons before the optical amplification and can also reduce the optical amplification noise. Regarding measurable samples, UC-TSIR can be applied to broadband MIR spectroscopy of condensed media by expanding the spectral bandwidth. For example, the spectral bandwidth can be larger than hundreds of cm^−1^ by using a few-mm-long PPLN crystal for upconversion. The foreseeing applications of the broadband UC-TSIR spectrometer are, for example, high-throughput single-cell analysis^42,43^ or accurate molecular fingerprinting of bio-molecules for health monitoring^8^. The concept of wavelength-conversion TSIR can also be applied to other wavelength regions where low-loss time-stretchers do not exist. Although the current UC-TSIR system can be operated in a laboratory only due to the bulky fs MIR-OPO, it can be portable by using a compact and stable MIR source such as fiber-based lasers^31,33^.

Finally, we discuss another spectroscopic aspect of UC-TSIR compared to FTIR, particularly the wavenumber calibration and the consequent accuracy. In UC-TSIR, it is necessary to calibrate the wavenumber scale by measuring the dispersion of optical fibers. We use a molecular absorption spectrum for the calibration, which limits the wavenumber accuracy. On the other hand, FTIR is capable of accurate interferometric calibration, e.g., with a He–Ne laser in a Michelson-type FTIR. DCS can provide extremely high accuracy given the nature of frequency combs with the level of atomic clocks. Therefore, Fourier-transform spectroscopy has an advantage in highly accurate precision spectroscopy, which is not within the main scope of the high-speed TSIR because precision measurement demands an extremely high SNR with a long measurement time.

In summary, we demonstrated UC-TSIR spectroscopy and showed high-speed broadband MIR spectroscopy of gas-phase molecules at an unprecedented level. By taking advantage of the superiority of the optical components and devices in the telecommunication region, we significantly improved the measurable number of spectral elements and the spectral resolution by maintaining a high SNR and measurement speed. The UC-TSIR spectrometer could enable various applications, particularly measurement of complex irreversible phenomena at a high temporal resolution^5,7,22^, statistical analysis of a large number of high contents spectral data^9,42,43^, and broadband hyperspectral image acquisition at a high frame rate^44–46^. It can also be applicable to other sensing techniques, such as MIR optical coherence tomography^47^ for 3D deep-inside profiling of highly scattering media.

## Materials and methods

### Light sources

We use a homemade fs MIR-OPO pumped by an 80-MHz Ti:Sapphire mode-locked laser (Maitai, Spectra-Physics) as a broadband MIR light source. The OPO generates MIR idler pulses with an average power of around 100 mW. In our experiment, the center wavenumber is adjusted to 2880 cm^−1^ (3.47 µm), whose −10-dB spectral bandwidth is about 235 cm^−1^, as shown in Fig. S1a. The MIR pulses are coupled into an InF_3_ single-mode fiber using an aspheric lens for spatial mode cleaning. A MIR bandpass filter with a bandwidth of 49 cm^−1^ is installed before the fiber-coupling to suppress undesired nonlinear optical effects in the optical fiber and the PPLN waveguide for upconversion. The fiber-output MIR pulses are collimated with a collimator and pass through a sample. We use gaseous CH_4_ molecules as a sample (CH_4_-T(25×5)-10-MgF_2_, Wavelength References) whose path length and pressure are 5 cm and 10 Torr, respectively. The pulses are tailored to be linearly polarized with a quarter-wave and a half-wave plate (QWP and HWP) and pass through a wire-grid polarizer and a dichroic mirror.

For upconverting the MIR pulses to the NIR region, we use a continuous-wave 1.064-µm distributed Bragg reflector (DBR) laser (PH1064DBR200BF, Photodigm) with a linewidth of 10 MHz. The CW laser passing through a fiber isolator is amplified with a homemade Yb-doped fiber amplifier and collimated with a collimator. The output beam goes through a free-space isolator, an HWP, and a 1-µm long-pass filter (LPF). The beam diameter and divergence are adjusted with a relay-lens pair. The beam is collinearly combined with the MIR pulses with the dichroic mirror.

### Upconversion

The combined MIR and NIR beams are focused onto a 20-mm-long PPLN waveguide (WD-3418-000-A-C-C-TEC, NTT Electronics) using a ZnSe aspheric lens with a focal length of 4.8 mm. The average power of the MIR and NIR beams measured before the ZnSe lens are a few mW and around 400 mW, respectively. The average power of the MIR pulses is intentionally decreased before coupling into the InF_3_ fiber to suppress the undesired nonlinear optical effects in the fiber and the PPLN waveguide. Due to the DFG process in the PPLN waveguide with a poling period of 28.6 µm, a part of the 3.4-µm MIR pulses is converted to 1.5-µm NIR pulses. As shown in Fig. S1a, the center wavelength of the upconverted NIR pulses can be tuned by controlling the temperature of the PPLN crystal with a Peltier temperature controller. The NIR pulses with an average power of a few µW are collected using an aspheric lens and pass through a 1.5-µm LPF and an HWP. Then, the NIR pulses are coupled into a single-mode fiber with another aspheric lens, whose coupling efficiency is around 0.65.

### Amplification and pulse pick

The fiber-coupled NIR pulses are amplified using an Er-doped fiber amplifier with a gain up to ~30 dB (EDFA100P, Thorlabs) and sent to a time stretcher. In large stretching cases, a pulse picker is implemented before the stretcher so that the adjacent NIR pulses do not temporally overlap each other. The pulse picker consists of a 200-MHz acousto-optic modulator (AOM) (T-M200-0.1C2J-3-F2P, Gooch&Housego) and a homemade RF driver. The RF driver generates 7-ns burst pulses with a carrier frequency of 200 MHz at a repetition rate of 10 MHz. The intensity modulation of the AOM generates NIR pulses at 10 MHz from the 80-MHz pulses. The full width at half maximum of the intensity modulation is 9 ns, which is determined by the width of the RF burst pulse and the rise and fall time of the AOM.

### Time-stretch

For time-stretching, we use DCF modules (AD-SM-C-120-FC/APC-3C/3B-10, YOFC), whose dispersion parameter and insertion-loss are −0.2 ns nm^−1^ km^−1^ and −0.7 dB km^−1^, respectively. The total length of DCF with the modules is 10–30 km, which corresponds to dispersion from −2 to −6 ns nm^−1^. The dispersion of −12 ns nm^−1^ is achieved by the double-pass geometry of the 30-km DCF realized by implementing a fiber retro-reflector and a circulator. In large stretching cases, a Raman amplifier is additionally implemented to provide sufficient signals for detection. The Raman amplifier consists of a fiber-coupled 1.455-µm fiber-Bragg-grating (FBG)-stabilized laser (PL-FP-1455-A-A81-SA, LD-PD) with an average power of around 400 mW. The beam is coupled to the DCFs with wavelength division multiplexers (WDMs). A bidirectional pumping configuration is adopted to suppress the NIR pulses’ self-phase modulation (SPM) in the DCFs while keeping the high Raman gain. The pump beam is separated by a 75:25 fiber beam splitter for bidirectional pumping. A detailed discussion about the Raman amplifier is described in Supplementary Note 3.

### Detection

The temporally stretched NIR pulses are collimated with a collimator, spectrally filtered with 1.55-µm bandpass filters (BPF) with a bandwidth of 50 cm^−1^ (12 nm), and coupled into a fiber with another collimator. The spectral filtering avoids temporal overlap of adjacent TSIR spectra and rejects amplifier noise outside the spectral bandwidth of the upconverted NIR pulses. The NIR pulses are detected with an AC-coupled 11-GHz InGaAs photodetector (RXM10AF, Thorlabs) and digitized with a high-speed 16-GHz oscilloscope (WaveMaster 816Zi-B, Teledyne LeCroy) at a sampling rate of 80 GSamples s^−1^.

### Retrieval of transmittance spectra

The measurement process of a TSIR waveform is written as

$$I = {{{\mathcal{L}}}}\left[ {\left| {{{{\mathcal{F}}}}^{ - 1}\left[ {D\;\sqrt {GB} \exp \left( { - i\;{{{\mathcal{K}}}}\left[ { - \frac{{\ln \left( {GB} \right)}}{2}} \right]} \right)} \right]} \right|^2} \right]$$

where $B \in {\Bbb R}\left[ {0,1} \right]$ is a frequency-domain transmittance spectrum, $G \in {\Bbb R}\left[ {0,} \right.\left. \infty \right)$is a baseline spectrum, ${{{\mathcal{K}}}}\left[ \cdot \right]$ denotes the K–K transformation, $D \in {\Bbb C}$ is the dispersion term (consisting of GDD and TOD), ${{{\mathcal{F}}}}^{ - 1}$ denotes the inverse Fourier transform, ${{{\mathcal{L}}}}\left[ \cdot \right]$ denotes low-pass filtering, which is determined by the measured low-pass filter function (Fig. S5), and $I \in {\Bbb R}\left[ {0,} \right.\left. \infty \right)$ is a measured time-domain TSIR waveform, respectively. The cut-off frequency of the low-pass filter is 11 GHz. The GDD and TOD values used in the algorithm are 2545 ps^2^ and −16 ps^3^ for a 10-km DCF, 7664 ps^2^ and -48 ps^3^ for a 30-km DCF, and 15,326 ps^2^ and −96 ps^3^ for a 60-km DCF.

We simultaneously estimate the frequency-domain transmittance spectrum *B* and the baseline spectrum *G* by using an iterative GD algorithm called Adam^38^. Before starting the iterations, the measured time-domain TSIR waveform *I* is truncated by a box-car function to restrict the frequency range for the estimation. In each iteration, we apply constraints of the sparsity and the transmittance range [0,1] to *B* with the alternating direction method of multiplier^48^ and smooth *G* with the SG filter^37^. After the iterations, the spectral resolution of *B* is finally adjusted to the achievable resolution (considering the width of the impulse response function) by applying the triangular apodization in the time domain. The yielded spectrum is plotted as *B*.

## Supplementary information

Supplementary Information

## Data Availability

The data provided in the manuscript are available from the corresponding author upon reasonable request.

## References

[1] Griffiths, P. R. & De Haseth, J. A. *Fourier Transform Infrared Spectrometry* 2nd edn. (John Wiley & Sons, Hoboken, NJ, 2007).

[2] Cossel KC (2017). Gas-phase broadband spectroscopy using active sources: progress, status, and applications. J. Opt. Soc. Am. B.

[3] Ycas G (2020). Compact mid-infrared dual-comb spectrometer for outdoor spectroscopy. Opt. Express.

[4] Giorgetta FR (2021). Open-path dual-comb spectroscopy for multispecies trace gas detection in the 4.5–5 µm spectral region. Laser Photonics Rev..

[5] Pinkowski NH (2020). Dual-comb spectroscopy for high-temperature reaction kinetics. Meas. Sci. Technol..

[6] Makowiecki AS (2021). Mid-infrared dual frequency comb spectroscopy for combustion analysis from 2.8 to 5 μm. Proc. Combust. Inst..

[7] Klocke JL (2018). Single-shot sub-microsecond mid-infrared spectroscopy on protein reactions with quantum cascade laser frequency combs. Anal. Chem..

[8] Pupeza I (2020). Field-resolved infrared spectroscopy of biological systems. Nature.

[9] Huber M (2021). Stability of person-specific blood-based infrared molecular fingerprints opens up prospects for health monitoring. Nat. Commun..

[10] Liang QZ (2021). Ultrasensitive multispecies spectroscopic breath analysis for real-time health monitoring and diagnostics. Proc. Natl Acad. Sci. USA.

[11] Liang, Q. Z. et al. Frequency comb and machine learning-based breath analysis for COVID-19 classification. Preprint at 10.48550/arXiv.2202.02321 (2022).

[12] Fleisher AJ (2014). Mid-infrared time-resolved frequency comb spectroscopy of transient free radicals. J. Phys. Chem. Lett..

[13] Nugent-Glandorf L (2012). Mid-infrared virtually imaged phased array spectrometer for rapid and broadband trace gas detection. Opt. Lett..

[14] Coddington I, Newbury N, Swann W (2016). Dual-comb spectroscopy. Optica.

[15] Muraviev AV (2018). Massively parallel sensing of trace molecules and their isotopologues with broadband subharmonic mid-infrared frequency combs. Nat. Photonics.

[16] Ycas G (2018). High-coherence mid-infrared dual-comb spectroscopy spanning 2.6 to 5.2 μm. Nat. Photonics.

[17] Timmers H (2018). Molecular fingerprinting with bright, broadband infrared frequency combs. Optica.

[18] Villares G (2014). Dual-comb spectroscopy based on quantum-cascade-laser frequency combs. Nat. Commun..

[19] Sterczewski LA (2019). Mid-infrared dual-comb spectroscopy with interband cascade lasers. Opt. Lett..

[20] Yu MJ (2018). Silicon-chip-based mid-infrared dual-comb spectroscopy. Nat. Commun..

[21] Yan M (2017). Mid-infrared dual-comb spectroscopy with electro-optic modulators. Light Sci. Appl..

[22] Yu MJ (2019). Microfluidic mid-infrared spectroscopy via microresonator-based dual-comb source. Opt. Lett..

[23] Süss B, Ringleb F, Heberle J (2016). New ultrarapid-scanning interferometer for FT-IR spectroscopy with microsecond time-resolution. Rev. Sci. Instrum..

[24] Hashimoto K, Badarla VR, Ideguchi T (2021). High-speed Fourier-transform infrared spectroscopy with phase-controlled delay line. Laser Photonics Rev..

[25] Tsai T, Wysocki G (2010). External-cavity quantum cascade lasers with fast wavelength scanning. Appl. Phys. B.

[26] Loparo ZE (2019). Acousto-optically modulated quantum cascade laser for high-temperature reacting systems thermometry. Opt. Lett..

[27] Lyakh A (2015). External cavity quantum cascade lasers with ultra rapid acousto-optic tuning. Appl. Phys. Lett..

[28] Childs DTD (2015). Sensitivity advantage of QCL tunable-laser mid-infrared spectroscopy over FTIR spectroscopy. Appl. Spectrosc. Rev..

[29] Kawai A (2020). Time-stretch infrared spectroscopy. Commun. Phys..

[30] Xu YQ, Murdoch SG (2019). Real-time spectral analysis of ultrafast pulses using a free-space angular chirp-enhanced delay. Opt. Lett..

[31] Johnson TA, Diddams SA (2012). Mid-infrared upconversion spectroscopy based on a Yb: fiber femtosecond laser. Appl. Phys. B.

[32] Neely TW (2012). Broadband mid-infrared frequency upconversion and spectroscopy with an aperiodically poled LiNbO_3_ waveguide. Opt. Lett..

[33] Chen, Z. J., Hänsch, T. W. & Picqué, N. Upconversion mid-infrared dual-comb spectroscopy. Preprint at 10.48550/arXiv.2003.06930 (2020).

[34] Liu, M. C. et al. A. Mid-infrared cross-comb spectroscopy. Preprint at 10.48550/arXiv.2107.08333 (2021).

[35] Chou J, Solli DR, Jalali B (2008). Real-time spectroscopy with subgigahertz resolution using amplified dispersive Fourier transformation. Appl. Phys. Lett..

[36] Goda K, Jalali B (2013). Dispersive Fourier transformation for fast continuous single-shot measurements. Nat. Photonics.

[37] Savitzky A, Golay MJE (1964). Smoothing and differentiation of data by simplified least squares procedures. Anal. Chem..

[38] Kingma, D. P. & Ba, J. ADAM: a method for stochastic optimization. In Benigo, Y. & LeCun, Y. (eds) *Proc. 3rd International Conference on Learning Representations* (ICLR, San Diego, 2015).

[39] Gordon IE (2017). The HITRAN2016 molecular spectroscopic database. J. Quant. Spectrosc. Radiat. Transf..

[40] Dougakiuchi T (2021). Ultimate response time in mid-infrared high-speed low-noise quantum cascade detectors. Appl. Phys. Lett..

[41] Loparo ZE (2018). Shock tube demonstration of acousto-optically modulated quantum cascade laser as a broadband, time-resolved combustion diagnostic. J. Energy Resour. Technol..

[42] Nitta N (2020). Raman image-activated cell sorting. Nat. Commun..

[43] Hiramatsu K (2019). High-throughput label-free molecular fingerprinting flow cytometry. Sci. Adv..

[44] Camp CH, Cicerone MT (2015). Chemically sensitive bioimaging with coherent Raman scattering. Nat. Photonics.

[45] Shi LX (2020). Mid-infrared metabolic imaging with vibrational probes. Nat. Methods.

[46] Khan FU, Guarnizo G, Martín-Mateos P (2020). Direct hyperspectral dual-comb gas imaging in the mid-infrared. Opt. Lett..

[47] Israelsen NM (2019). Real-time high-resolution mid-infrared optical coherence tomography. Light Sci. Appl..

[48] Chan SH, Wang XR, Elgendy OA (2017). Plug-and-play ADMM for image restoration: fixed-point convergence and applications. IEEE Trans. Comput. Imaging.

