# Supplementary material for: Upconversion time-stretch infrared spectroscopy
Source: Light Sci Appl. 2023 Mar 4;12:48. doi: 10.1038/s41377-023-01096-4 (PMC9984475; doi:10.1038/s41377-023-01096-4)
Supplement: Supplementary file 1 — Supplementary Information [file 41377_2023_1096_MOESM1_ESM.pdf]

Supplementary Information for
Upconversion time-stretch infrared spectroscopy

Kazuki Hashimoto[1], Takuma Nakamura[1], Takahiro Kageyama[2], Venkata Ramaiah Badarla[1], Hiroyuki Shimada[1], Ryoichi Horisaki[3], and Takuro Ideguchi[1,2,*]

[1] Institute for Photon Science and Technology, The University of Tokyo, Tokyo, 113-0033, Japan

[2] Department of Physics, The University of Tokyo, Tokyo, 113-0033, Japan

[3] Department of Information Physics and Computing, The University of Tokyo, Tokyo, 113-8656, Japan

*ideguchi@ipst.s.u-tokyo.ac.jp

Supplementary Note 1: Detailed schematic of upconversion TSIR

A detailed schematic of upconversion time-stretch infrared spectroscopy (UC-TSIR) is shown in Figure S1a. Three configurations of the time stretchers with different amounts of dispersion are depicted in Figure S1b.

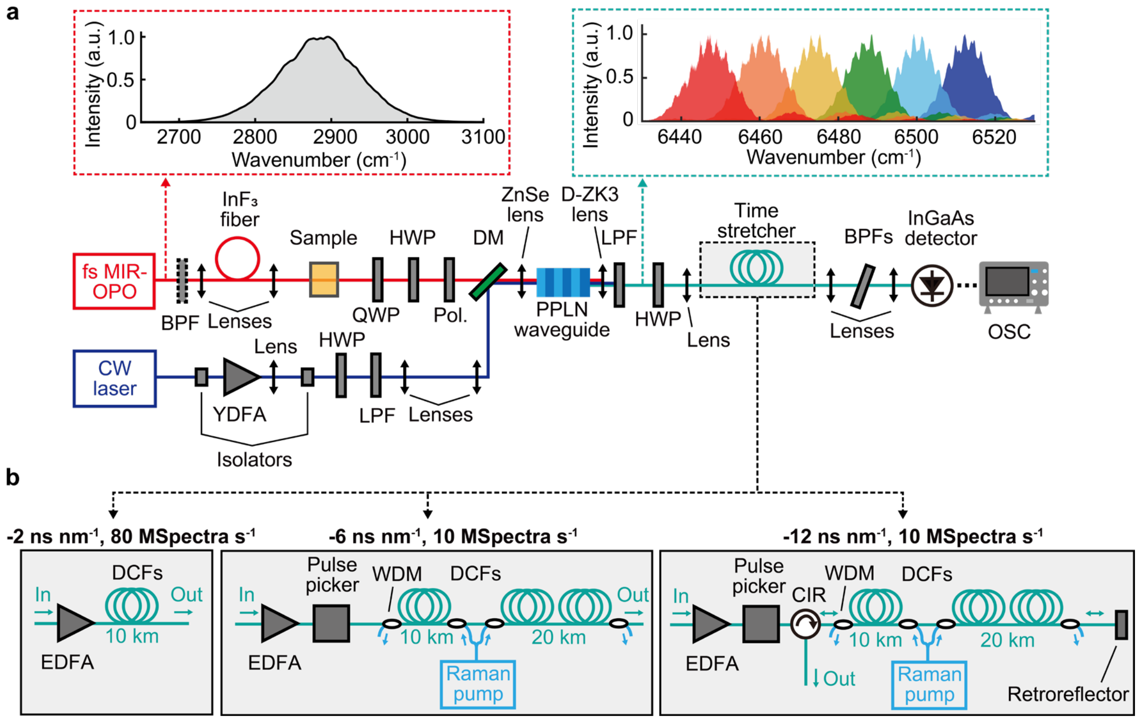

Figure S1: a Detailed schematic of upconversion TSIR. The insets show a mid-infrared (MIR) spectrum generated by a fs MIR optical parametric oscillator (MIR-OPO) (left) and upconverted near-infrared (NIR) spectra generated at different periodically-poled lithium niobate (PPLN) temperatures (13 - 63°C) (right). BPF: band-pass filter, QWP: quarter-wave plate, HWP: half-wave plate, Pol.: polarizer, DM: dichroic mirror, YDFA: Yb-doped fiber amplifier, LPF: long-pass filter, OSC: oscilloscope. **b** Time-stretcher configurations. EDFA: Er-doped fiber amplifier, DCF: dispersion-compensating fiber, WDM: wavelength division multiplexer, CIR: circulator.

Supplementary Note 2: Nonlinear optical effects in a PPLN waveguide

We evaluate the undesired nonlinear optical effects of MIR pulses occurring in a PPLN waveguide. Figure S2 shows a part of upconverted CH_4 spectra with an average power of MIR pulses illuminated onto the PPLN waveguide of 3.6, 2.7, 1.8, 1.4, and 0.9 mW. The spectra are measured with an optical spectrum analyzer (OSA) with a spectral resolution of 0.08 cm^{-1} (0.02 nm). The average power of a 1-µm continuous-wave (CW) laser for upconversion is fixed at 300 mW. As shown in the figure, the absorption lines on the upconverted spectrum distort when the average power of the MIR pulses is high. We observe that this effect does not depend on the input power of the 1-µm CW laser but on the input power and the spectral bandwidth (pulse duration) of the MIR pulses. Therefore, it is likely caused by the nonlinear optical effects (e.g., self-phase modulation (SPM)) of the MIR pulses in a PPLN waveguide. To avoid the spectral distortions, we adjust the input MIR average power to less than 1.4 mW (pulse energy of 17.5 pJ).

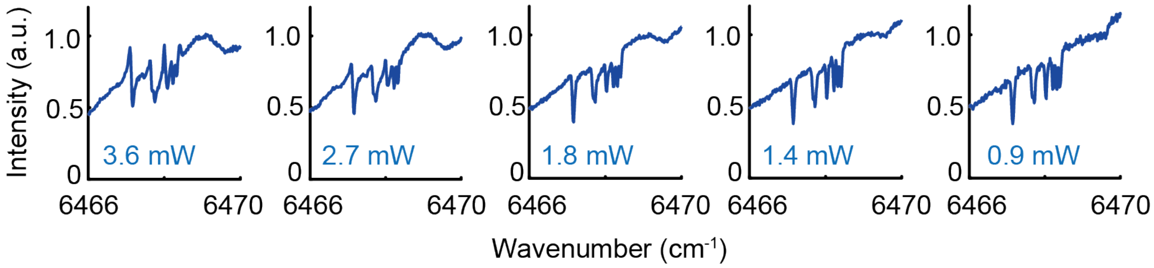

Figure S2: A part of the upconverted CH_4 spectra measured with an OSA at a spectral resolution of 0.08 cm^{-1}. They are obtained at the average power of the input MIR pulses illuminated onto the PPLN waveguide of 3.6, 2.7, 1.8, 1.4, and 0.9 mW, respectively.

Supplementary Note 3: Nonlinear optical effects in DCFs

We also observe undesired nonlinear effects on the upconverted NIR pulses propagating in a long DCF. Figure S3a shows a part of the upconverted CH_4 spectra measured after a 30-km DCF (group delay dispersion (GDD): 7,664 ps^2, third-order dispersion (TOD): -48 ps^3) that also works as a forward-pump Raman amplifier pumped with an average power of 310 or 150 mW. The repetition rate and the average power of the input NIR pulses are 10 MHz and ~20 µW (pulse energy of 2 pJ), respectively. The spectra are measured with an OSA with a spectral resolution of 0.08 cm^{-1}. As shown in the figure, the CH_4 absorption lines distort when the average pump power of the Raman amplifier is high.

To evaluate the distortion of the absorption line profile of the NIR spectra after propagating a long DCF, we simulate the SPM effects of the NIR pulses in the DCF with Raman amplification using the split-step Fourier method[1]. The calculation parameters are set to resemble the experiment shown in Figure S3a. We assume a transform-limited 1-ps gaussian-shaped NIR pulse with a pulse energy of 2 pJ (corresponding to an average power of 20 µW at a repetition rate of 10 MHz), which interacts with molecules that have an absorption line with a linewidth of 0.01 cm^{-1} (260 MHz). The pulse propagates a 30-km DCF with a forward-pump Raman amplifier (group-velocity dispersion (GVD): 255 ps^2 km^{-1}, TOD per unit length: -1.6 ps^3 km^{-1}, nonlinear coefficient: 5.7 W^{-1} km^{-1}, signal insertion loss: -0.7 dB km^{-1},

Raman-pump loss: -1.3 dB km^{-1}, Raman-gain coefficient: 4.7 W^{-1} km^{-1}) and is measured with a spectrometer with a spectral resolution of 0.05 cm^{-1}. Figure S3b shows a part of the simulated NIR spectra after propagating the DCF with various Raman pump powers (0-400 mW), demonstrating the absorption-line distortion caused by the SPM of the NIR pulses with a high peak power gained by the Raman amplifier. Figure S3c shows the peak power of the NIR pulses against the fiber length with the Raman pump powers of 0, 100, 200, 300, and 400 mW. These simulations show that a 10-km propagation with a peak power larger than 10^{-2} W causes the line-profile distortion. A backward pump configuration could alleviate the distortion.

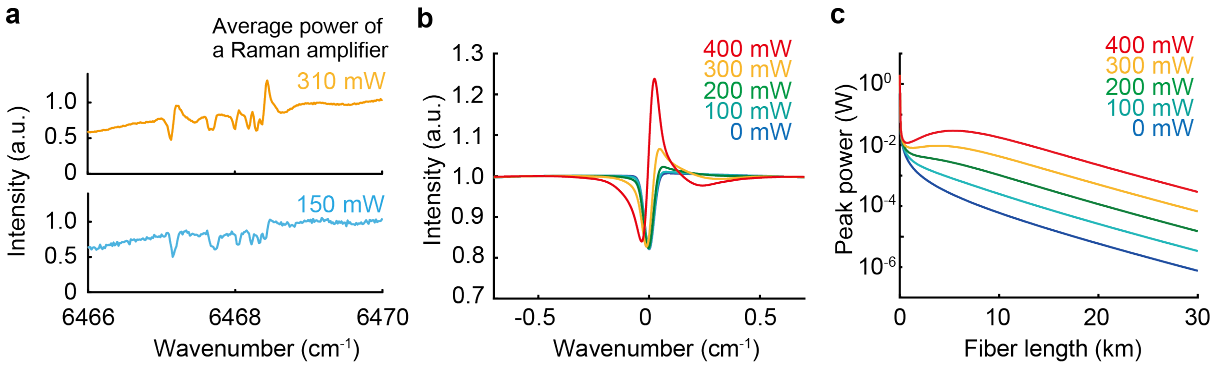

Figure S3: Line-profile distortion due to the SPM of a NIR pulse in a 30-km DCF with a forward-pump Raman amplification. **a** Comparison of a part of the upconverted CH_4 spectra amplified with an average pump power of 310 mW (upper) and 150 mW (lower). The spectra are measured with an OSA with a spectral resolution of 0.08 cm^{-1}. **b** A part of the simulated spectra of a NIR pulse propagating after a 30-km DCF with average pump powers of 0, 100, 200, 300, and 400 mW. **c** Peak power of the simulated NIR pulse against the fiber length with pump powers of 0, 100, 200, 300, and 400 mW.

To avoid the distortion while keeping the high signal-to-noise ratio (SNR), we implement a bidirectional-pump Raman amplifier, as illustrated in Figure S1b. Figure S4a shows a simulation result of the peak power of a NIR pulse against the fiber length in the bidirectional-pump Raman amplification, where the first part (0-10 km) is amplified with a 100-mW backward pump and the second part (10-30 km) with a 250-mW forward pump. The bidirectional-pump amplifier can keep the NIR peak power less than 10^{-2} W for long-distance propagation. Figure S4b shows a part of the spectrum with a NIR pulse propagating after the 30-km DCF with the bidirectional-pump Raman amplification, showing the distortion-less time-stretching. Finally, we experimentally demonstrate the bidirectional-pump Raman amplification. We measure upconverted CH_4 spectra of a NIR pulse propagating after a 30-km DCF with a bidirectional-pump Raman amplification (100-mW backward pump for the first 10 km and 250-mW forward pump for the second 20 km) using an OSA at a spectral resolution of 0.08 cm^{-1} (Figure S4c), showing no line-profile distortions. It also keeps a high average power of the NIR pulses at tens of µW (a few pJ) after the DCF.

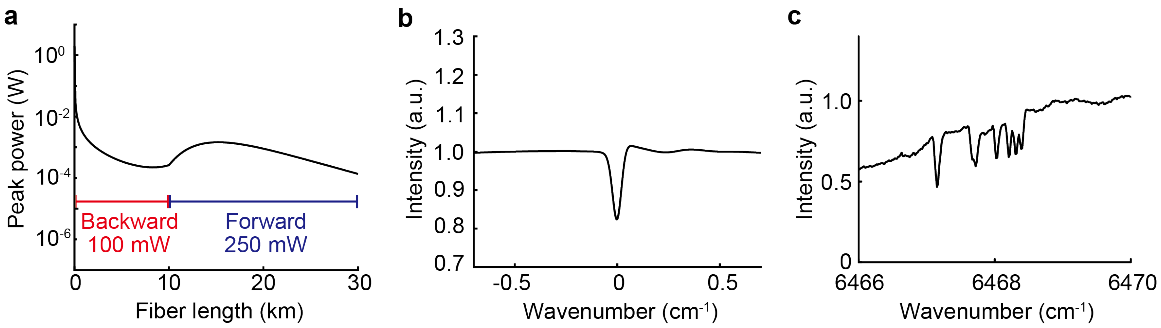

Figure S4: SPM effects of a NIR pulse in a 30-km DCF with a bidirectional-pump Raman amplification. **a** Simulated peak power of a NIR pulse against the fiber length with a bidirectional Raman pump (100-mW backward pump for the first 10 km and 250-mW forward pump for the second 20 km). **b** A part of the simulated spectrum of a NIR pulse propagating after a 30-km DCF with the bidirectional-pump Raman amplification. **c** A part of the experimentally measured upconverted CH_4 spectrum with the bidirectional-pump Raman amplification. It is measured with an OSA with a spectral resolution of 0.08 cm^{-1}.

Supplementary Note 4: Characterization of the InGaAs photodetector

We evaluate the frequency response of the InGaAs photodetector used in our experiment. Figure S5a shows the impulse response obtained by measuring an unstretched NIR pulse with the detector, showing the full width at half maximum (FWHM) of 49 ps. Figure S5b shows the frequency response of the detector obtained by Fourier-transforming the train of the temporal impulse responses. The -3dB bandwidth of the detector is evaluated as 11 GHz.

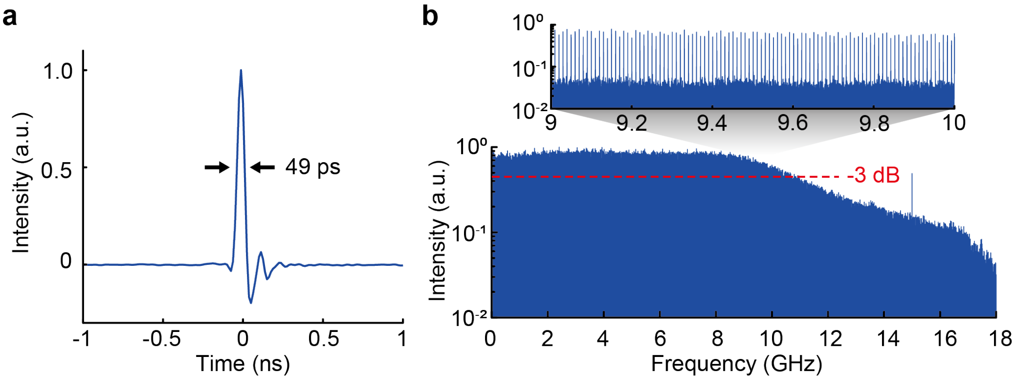

Figure S5: Characterization of the InGaAs photodetector used in our experiment. **a** An impulse response of the detector. The FWHM is 49 ps. **b** Frequency response of the detector. The -3dB bandwidth is 11 GHz.

Supplementary Note 5: Theoretical description of a TSIR spectrum

We theoretically describe a TSIR waveform to show why the absorption lines on the spectrum become dispersive. In this description, we only consider GDD as a dispersion for time-stretching for simplicity. We add GDD to an input electric field, $E_{\mathrm{in}}(\omega)$, in the frequency domain as

$$E_{\mathrm{out}}(\omega) = E_{\mathrm{in}}(\omega)\exp\left\{i\frac{\beta_2 L}{2}(\omega-\omega_0)^2\right\} \tag{S1}$$

where ω, ω_0, β_2, and L denote an angular frequency, a center angular frequency, GVD, and a fiber length, respectively. $E_{\text{in}}(\omega)$ and $\exp\left\{i\frac{\beta_2 L}{2}(\omega-\omega_0)^2\right\}$ correspond to $\sqrt{GB}\exp\left(-i\,\mathcal{K}\left[-\frac{\ln(GB)}{2}\right]\right)$ and D in Methods in the main text, respectively. The time-domain electric field, $e_{\text{out}}(t)$, is written as

$$\begin{aligned} e_{\text{out}}(t) &= \mathcal{F}^{-1}[E_{\text{out}}(\omega)] \\ &= \mathcal{F}^{-1}\left[E_{\text{in}}(\omega)\exp\left\{i\frac{\beta_2 L}{2}(\omega-\omega_0)^2\right\}\right] \\ &= \mathcal{F}^{-1}[E_{\text{in}}(\omega)]\otimes\mathcal{F}^{-1}\left[\exp\left\{i\frac{\beta_2 L}{2}(\omega-\omega_0)^2\right\}\right] \end{aligned} \tag{S2}$$

where t, $\mathcal{F}^{-1}$, and $\otimes$ denote time, the symbols of operations of inverse Fourier transform and convolution, respectively. $\mathcal{F}^{-1}[E_{\text{in}}(\omega)]$ and $\mathcal{F}^{-1}\left[\exp\left\{i\frac{\beta_2 L}{2}(\omega-\omega_0)^2\right\}\right]$ are individually described as

$$\begin{aligned} \mathcal{F}^{-1}\{E_{\text{in}}(\omega)\} &= \frac{1}{2\pi}\int\{E_{\text{in}}(\omega)\}\exp(i\omega t)d\omega \\ &= e_{\text{in}}(t) \end{aligned} \tag{S3}$$

and

$$\begin{aligned} \mathcal{F}^{-1}\left[\exp\left\{i\frac{\beta_2 L}{2}(\omega-\omega_0)^2\right\}\right] &= \frac{1}{2\pi}\int\left[\exp\left\{i\frac{\beta_2 L}{2}(\omega-\omega_0)^2\right\}\right]\exp(i\omega t)d\omega \\ &= \frac{1}{\sqrt{2\pi\beta_2 L}}\exp\left\{i\left(\frac{\beta_2 L}{2}\omega_0^2+\frac{\pi}{4}\right)\right\}\exp\left\{-i\frac{(t-\beta_2 L\omega_0)^2}{2\beta_2 L}\right\} \end{aligned} \tag{S4}$$

respectively, where $e_{\text{in}}(t)$ denotes the time-domain input electric field. Then, $e_{\text{out}}(t)$ is described as

$$\begin{aligned} e_{\text{out}}(t) &= e_{\text{in}}(t)\otimes A\exp\left\{-i\frac{(t-\beta_2 L\omega_0)^2}{2\beta_2 L}\right\} \\ &= A\int e_{\text{in}}(t')\exp\left\{-i\frac{(t-\beta_2 L\omega_0-t')^2}{2\beta_2 L}\right\}dt' \end{aligned} \tag{S5}$$

where t' denotes time, and A is defined as $A=\frac{1}{\sqrt{2\pi\beta_2 L}}\exp\left\{i\left(\frac{\beta_2 L}{2}\omega_0^2+\frac{\pi}{4}\right)\right\}$. Using the variable, $\tau=t-\beta_2 L\omega_0$, Equation (S5) is rewritten as

$$e_{\text{out}}(\tau) = A\int e_{\text{in}}(t')\exp\left\{-i\frac{(\tau-t')^2}{2\beta_2 L}\right\}dt' \tag{S6}$$

Therefore, a TSIR waveform, $I(\tau)$, is expressed as

$$I(\tau) = |e_{\text{out}}(\tau)|^2 \tag{S7}$$

$$= |A|^2 \left| \int e_{\text{in}}(t') \exp\left\{-i \frac{(\tau - t')^2}{2\beta_2 L}\right\} dt' \right|^2$$

$$= |A|^2 \left| \int e_{\text{in}}(t') \exp\left(-i \frac{t'^2}{2\beta_2 L}\right) \exp\left(i \frac{\tau}{\beta_2 L} t'\right) dt' \right|^2$$

When $\left|\frac{t'^2}{2\beta_2 L}\right| \ll 1$, $I(\tau)$ is approximately written as

$$\begin{aligned} I(\tau) &\approx |A|^2 \left| \int e_{\text{in}}(t') \exp\left\{-i\left(-\frac{\tau}{\beta_2 L}\right) t'\right\} dt' \right|^2 \\ &= |A|^2 \left| \int e_{\text{in}}(t') \exp(-i\omega' t') dt' \right|^2 \\ &\propto |\mathcal{F}\{e_{\text{in}}(t')\}|^2 \\ &= |E_{\text{in}}(\omega')|^2 \end{aligned} \qquad \text{(S8)}$$

where $\mathcal{F}$ denotes the symbol of Fourier transform operation, and ω' is defined as $\omega' = -\frac{\tau}{\beta_2 L}$. In this regime, a TSIR waveform in the time domain represents the spectrum in the frequency domain (far-field regime). If the approximation is invalid, $e_{\text{in}}(t')$ is modulated by the term, $\exp\left(-i\frac{t'^2}{2\beta_2 L}\right)$, which yields the dispersive shape on the absorption lines (near-field regime).

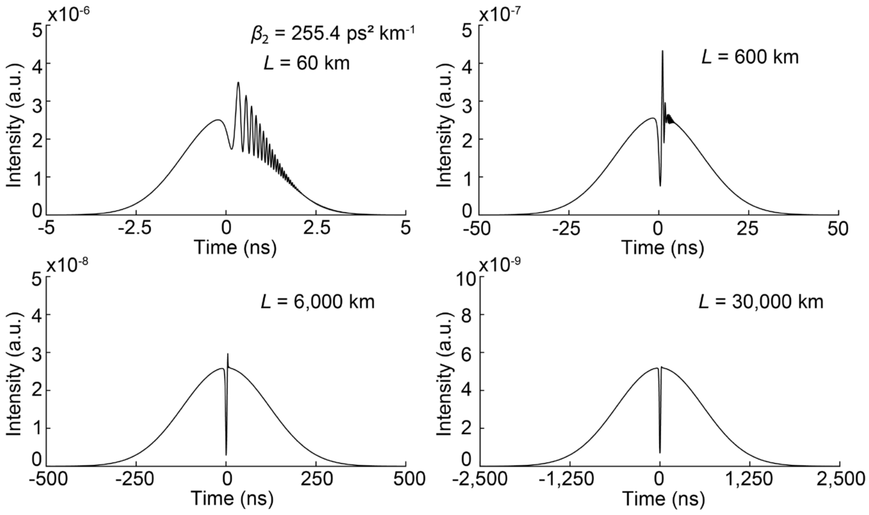

Figure S6: TSIR waveforms calculated with various amounts of dispersion. We calculate a 1-cm^{-1}-width gaussian-shape spectrum with a 0.01-cm^{-1}-width absorption line and temporally stretch it using fibers with a GVD of 255.4 ps^2 km^{-1} at four different lengths (60, 600, 6000, and 30000 km). β_2: GVD, L: fiber length.

We simulate TSIR waveforms with an absorption line using the above-described equations. Figure S6 shows TSIR waveforms calculated by adding various amounts of dispersions to a 1-cm^{-1}-width gaussian-shaped spectrum with a 0.01-cm^{-1}-width absorption line. As a dispersive medium, we assume a DCF with a GVD of 255.4 ps^2 km^{-1} at four different lengths (60, 600, 6,000, and 30,000 km). We find a 10^4-km DCF is necessary for a TSIR waveform to reach

the far-field regime, which is not experimentally realistic to be used. Therefore, it is practical to measure a TSIR waveform in the near-field regime with a moderate-length fiber and make a computational spectral correction. We note that the fiber length of 1 km is enough to reach the far-field regime when measuring condensed-phase infrared spectra with a physical linewidth of ~3 cm^{-1}.

Supplementary Note 6: Wavenumber instability of TSIR spectroscopy

We evaluate the wavenumber instability of TSIR spectroscopy by measuring the peak-position fluctuation of continuously measured single-shot TSIR spectra. The lower panel in Figure S7 shows the temporal variation of the TSIR spectra (0-9 μs) sequentially measured at 10 MSpectra s^{-1} with the 30-km DCF (total dispersion of -6 ns nm^{-1}). Each TSIR waveform is segmented, and the temporal position is calibrated with the peak at 43.11 ns (shown in the upper panel of Figure S7). The standard deviation of the peak positions around 56.16 ns is 9 ps (0.006 cm^{-1}), which is shorter than the sampling period of the oscilloscope (12.5 ps).

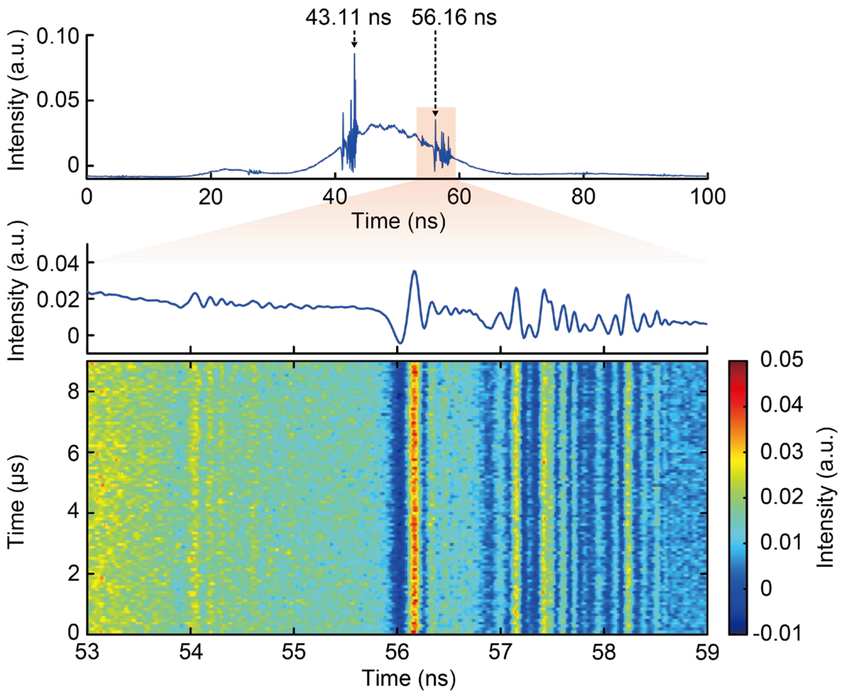

Figure S7: Wavenumber instability of TSIR spectroscopy. The upper and middle panels show the averaged TSIR spectrum measured at 10 MSpectra s^{-1} with the 30-km DCF and the enlarged view of the spectrum in the time frame of 53-59 ns, respectively. The lower panel represents the temporal variation of single-shot TSIR spectra in the time frame of 53-59 ns sequentially measured at 10 MSpectra s^{-1}.

Supplementary Note 7: Relative wavenumber accuracy of TSIR spectroscopy

We evaluate how the TOD affects the relative wavenumber accuracy of a retrieved spectrum. Figure S8a shows transmittance spectra of CH_4 molecules retrieved from 180-times averaged TSIR waveforms stretched with a dispersion of -12 ns nm^{-1}. The red plot represents the case where both GDD (=15,326 ps^2) and TOD (= -96 ps^3) values

are taken into account for the Gradient-descent (GD) algorithm, while the orange plot represents the case where only the GDD value is taken into account. The x-axis is the MIR-wavenumber downconverted from the NIR-wavenumber. As shown in the inset of the figure, the peak position of the orange plot is shifted by 0.027 cm^{-1} at 2,916 cm^{-1}. Figure S8b shows the difference in peak position (d_{wav}) against the peak positions of the calculated spectrum from the HITRAN database. The red dots represent the case where both GDD and TOD values are taken into account for the algorithm, while the orange dots represent the case where only the GDD value is taken into account. The absolute maxima of d_{wav} are 0.007 and 0.026 cm^{-1} in the former and the latter cases, respectively. It verifies taking into account the TOD is essential to improve the relative wavenumber accuracy.

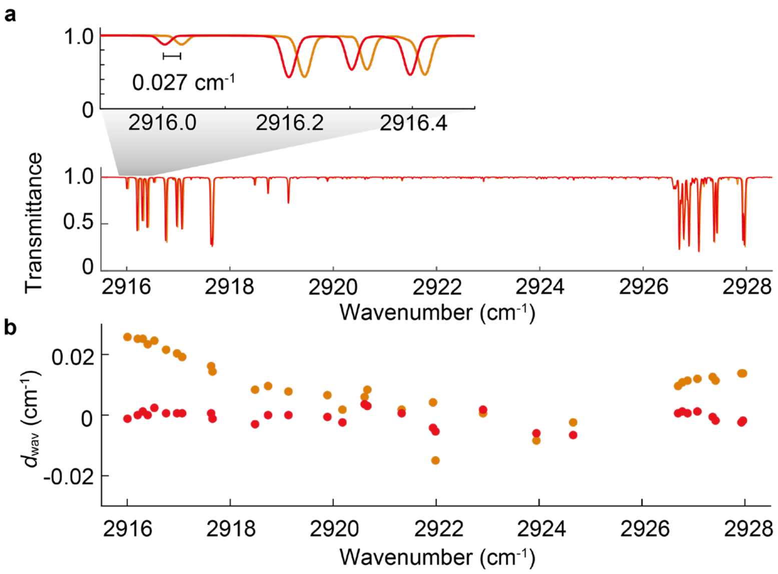

Figure S8: Relative wavenumber accuracy of TSIR spectroscopy. **a** Transmittance spectra of CH_4 molecules retrieved from TSIR waveforms stretched with a dispersion of -12 ns nm^{-1}. The red and orange plots represent the cases where the GD algorithm includes GDD and TOD values and a GDD value only, respectively. The inset shows a zoom-in view of the spectra. **b** Difference in absorption-peak position between the retrieved spectra and the calculated spectrum from the HITRAN database (d_{wav}). The red and orange dots represent the cases where the GD algorithm includes GDD and TOD values and a GDD value only, respectively.

Supplementary Note 8: SNR comparison between TSIR and FTIR spectra

We theoretically evaluate the SNR of a TSIR spectrum and compare it with that of an FTIR (FTIR: Fourier-transform infrared spectroscopy) spectrum. We individually derive the SNR of TSIR and FTIR spectra and then provide the ratio between them.

First, we derive the SNR of a TSIR spectrum. The SNR of a temporal intensity waveform, $I(t)$, is defined as

$$SNR_t(t) = \frac{I(t)}{\epsilon_t} \qquad \text{(S9)}$$

where t and ϵ_t denote time and noise (r.m.s. error)[2], respectively. For simplicity, we assume the white noise and a flat-top TSIR spectrum (constant signal intensity with respect to time) with a duty cycle of 1. The SNR of a TSIR

spectrum, SNR_{TS}, can be written as

$$SNR_{\mathrm{TS}} = \frac{P}{\sigma(P)\sqrt{f_{\mathrm{BW}}}} \tag{S10}$$

where P, $\sigma(P)$, and f_{BW} denote an average detection power, a system's overall noise-equivalent power (NEP), and an RF bandwidth of the system, respectively. The overall NEP depends on the average detection power because it contains various noises (e.g., detector noise, shot noise, or amplified spontaneous emission (ASE) noise). The RF bandwidth is mainly determined by the detector and the digitizer. Throughout this comparison, we set the sampling frequency is double the RF bandwidth, f_{BW}. In this condition, the SNR can be written as

$$SNR_{\mathrm{TS}} = \sqrt{\frac{2T}{N}}\frac{P}{\sigma(P)} \tag{S11}$$

where T and N denote a measurement time and the number of the sampling data points, respectively. Since the number of spectral elements, M, of TSIR measurement is equal to the number of the sampling data points, N, the SNR can be written as

$$SNR_{\mathrm{TS}} = \sqrt{\frac{2T}{M}}\frac{P}{\sigma(P)} \tag{S12}$$

The equation shows the SNR of a TSIR spectrum is proportional to the square root of the measurement time (spectral acquisition time) and inversely proportional to the square root of the number of spectral elements. If there is a duty cycle, $D_{\mathrm{TS}} = \frac{M}{N}\,(0 \le D_{\mathrm{TS}} \le 1)$, it may be written as

$$SNR_{\mathrm{TS}} = \sqrt{\frac{2T}{M}}\frac{1}{\sqrt{D_{\mathrm{TS}}}}\frac{P}{\sigma\left(\frac{P}{D_{\mathrm{TS}}}\right)} \tag{S13}$$

Next, we derive the SNR of an FTIR spectrum. We define the SNR of a spectral density, $B(f)$, as

$$SNR_f(f) = \frac{B(f)}{\epsilon_f} \tag{S14}$$

where f and ϵ_f denote frequency and noise, respectively. We first describe the SNR of a double-sided interferogram, $I(t)$, by the spectral density by following the procedure given in the literature[2]. The center burst of an interferogram, $I(0)$, can be written as

$$\begin{aligned} I(0) &= \sum_{j=1}^{N} B(f_j)\ \delta f \\ &= \bar{B} \cdot N \cdot \delta f \end{aligned} \tag{S15}$$

where $\bar{B}$ and δf denote the average spectral density and the spectral interval between adjacent spectral elements, respectively. Considering the energy conservation of Fourier transformation[2], the relation between the noises in the time and frequency domains is written as

$$(\epsilon_t)^2 \cdot \delta t \cdot N = \left(\epsilon_{f,\mathrm{tot}}\right)^2 \cdot \delta f \cdot N \tag{S16}$$

where δt and $\epsilon_{f,\mathrm{tot}}$ denote the sampling period in the time domain and the total noise in the frequency domain. Since only the real part of the Fourier transformation contributes to the FTIR noise ($\epsilon_f = \epsilon_{f,\mathrm{tot}}/\sqrt{2}$), ϵ_t can be written as

$$\epsilon_t = \sqrt{2}\epsilon_f\sqrt{\frac{\delta f}{\delta t}} \tag{S17}$$

Therefore, the SNR of the interferogram's center burst, $SNR_t(0)$, is written as

$$\begin{aligned} SNR_t(0) &= \frac{I(0)}{\epsilon_t} \\ &= \frac{\bar{B}\cdot N\cdot\delta f}{\sqrt{2}\epsilon_f\sqrt{\frac{\delta f}{\delta t}}} \\ &= \sqrt{\frac{N}{2}}\frac{\bar{B}}{\epsilon_f} \end{aligned} \tag{S18}$$

Here, we use the relation $\delta t\delta f = N^{-1}$. We can describe the SNR of an FTIR spectrum as

$$SNR_{\mathrm{FT}}(f) = \sqrt{\frac{2}{N}}\frac{B(f)}{\bar{B}}SNR_t(0) \tag{S19}$$

We calculate the SNR of the interferogram's center burst, $SNR_t(0)$. We assume that an interferogram with the visibility of 1 is AC-coupled and measured at a measurement time of T with the number of sampling data points of N. Since the two beams from the sample and reference arms constructively interfere at $t = 0$, $SNR_t(0)$ can be written as

$$\begin{aligned} SNR_t(0) &= \frac{P}{\sigma(P)\sqrt{f_{\mathrm{BW}}}} \\ &= \sqrt{\frac{2T}{N}}\frac{P}{\sigma(P)} \end{aligned} \tag{S20}$$

Considering the Nyquist-limited spectral measurement ($N/2 = M$) of a flat-top spectrum ($\bar{B} = B(f)$), the SNR of FTIR is described as

$$\begin{aligned} SNR_{\mathrm{FT}} &= \frac{1}{\sqrt{M}}SNR_t(0) \\ &= \frac{\sqrt{T}}{M}\frac{P}{\sigma(P)} \end{aligned} \tag{S21}$$

Now, we compare the SNR of TSIR (S12) and FTIR (S21) spectra. The SNR ratio of a TSIR spectrum and an FTIR spectrum, $R_{\mathrm{TS/FT}} = \frac{SNR_{\mathrm{TS}}}{SNR_{\mathrm{FT}}}$, is expressed as

$$R_{\mathrm{TS/FT}} = \sqrt{2M} \tag{S22}$$

It shows a TSIR spectrum has $\sqrt{2M}$-times higher SNR than an FTIR spectrum under the same measurement time. In other words, TSIR can take spectra with the same SNR at a $2M$-times higher spectral measurement rate than FTIR.

Finally, we compare the SNR of TSIR and FTIR, assuming that we can fully utilize the detector's dynamic range. Since half of the average detection power is used for the DC components of an FTIR interferogram, the dynamic-range-limited average detection power of FTIR is half of TSIR ($P_{\mathrm{TS}} = 2P_{\mathrm{FT}}$). To compare the SNR values under different average detection powers, we describe the overall NEP for each noise component as

$$\sigma_{\mathrm{TS(FT)}}\left(P_{\mathrm{TS(FT)}}\right) = \sqrt{NEP_{\mathrm{det}}{}^2 + \left(\sqrt{\frac{2P_{\mathrm{TS(FT)}}h\nu}{\eta}}\right)^2 + \left(\sqrt{(Amp)P_{\mathrm{TS(FT)}}{}^2}\right)^2} \tag{S23}$$

where $P_{\mathrm{TS(FT)}}$, NEP_{det}, h, ν, η, and Amp denote average detection power for TSIR (FTIR), detector NEP, the Planck constant, optical frequency, the quantum efficiency of a detector, and amplifier noise of an amplifier, respectively. The three terms, NEP_{det}, $\sqrt{\frac{2P_{\mathrm{TS(FT)}}h\nu}{\eta}}$, and $\sqrt{(Amp)P_{\mathrm{TS(FT)}}{}^2}$, correspond to the NEP dominated by the detector noise, the shot noise, and the amplifier noise, respectively. We assume the digitizer's dynamic range is sufficiently high and does not limit the SNR. With the relation $P_{\mathrm{TS}} = 2P_{\mathrm{FT}} = 2P$, the SNR ratio is expressed as

$$\begin{aligned} R_{\mathrm{TS/FT}} &= \frac{P_{\mathrm{TS}}}{P_{\mathrm{FT}}}\frac{\sigma_{\mathrm{FT}}(P_{\mathrm{FT}})}{\sigma_{\mathrm{TS}}(P_{\mathrm{TS}})}\sqrt{2M} \\ &= \left\{2\frac{\sigma_{\mathrm{FT}}(P)}{\sigma_{\mathrm{TS}}(2P)}\right\}\sqrt{2M} \\ &= \alpha\sqrt{2M} \end{aligned} \tag{S24}$$

The constant value α varies from 1 to 2 depending on the noise condition. For example, if the noise is dominated by the detector noise, the shot noise, or the amplifier noise, it becomes 2, $\sqrt{2}$, or 1, respectively.

Supplementary Note 9: SNR evaluation of upconversion TSIR spectroscopy

We evaluate the SNR of upconversion TSIR spectroscopy using transmittance TSIR waveforms measured at a rate of 10 MHz. The black dots in Figure S9 show the SNR of a single-shot TSIR waveform (measured with 30-km DCF) against the average power detected with an InGaAs detector. The average detection power is changed right before the photodetector. The SNR is evaluated from the standard deviation of a normalized waveform where no large peaks exist. It linearly increases against the average detection power at a lower power level while being saturated around ten above ~5 μW.

To compare them with theory, we also plot the calculated SNR. The SNR lines are calculated assuming a flat-top spectrum at a spectral measurement rate of 10 MHz, a duty cycle of 0.16 at FWHM, and an RF bandwidth of 11 GHz. We also assume the detector NEP of 15.5 pW $\mathrm{Hz}^{-\frac{1}{2}}$, the wavenumber of 6,471 cm^{-1}, and the detector quantum efficiency of 0.72. The blue line in the figure represents a detector-noise-limited SNR calculated from the NEP of the InGaAs photodetector and the digitizer, which linearly increases against the average detection power. It agrees well with the measured SNR at a lower average detection power than 5 μW. The green line represents the shot-noise-limited SNR of the current system, which is determined by the number of photons (corresponding to an average

power of 250 nW at 10 MHz) before the optical amplification with the EDFA. The measured SNR above 5 μW is slightly lower than the shot-noise-limited SNR. We note that the shot-noise-limited SNR described here, $SNR_{shot\text{-}UC}$, does not depend on the average detection power. We attribute the additional noise to the optical amplifier, which also gives a constant SNR against the average detection power with the photodetector. The orange line shows the upper limit of an input power where the detector linearly responds without saturation.

The SNR can be improved by making some modifications to the system. One can realize the detector-noise limited SNR up to ~90 at a higher average detection power above 5 μW by increasing the number of upconverted photons before the optical amplification, which improves the shot-noise-limited SNR and can also reduce the optical amplification noise. We expect the number of upconverted photons can increase by injecting a higher power of the 1-μm laser into the PPLN waveguide. There is a potential to increase the SNR further by using a lower-noise detector. The red line in Figure S9 represents the shot-noise-limited SNR determined by the number of photons captured by a photodetector, SNR_{shot}, which linearly increases against the square root of the average detection power.

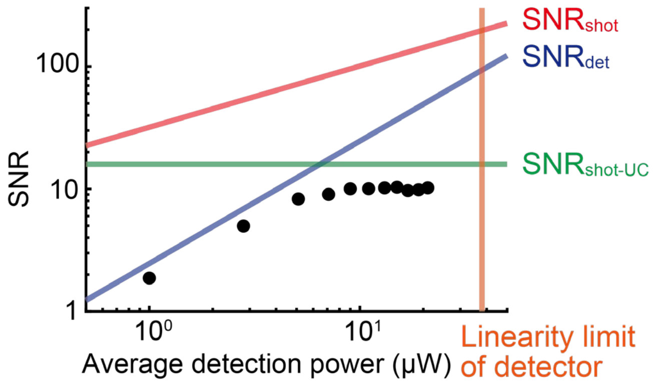

Figure S9: Single-shot SNR of a TSIR waveform against an average power captured by an InGaAs photodetector. The black dots represent the SNR of the single-shot normalized TSIR waveforms measured at 10 MSpectra s^{-1} with 30-km DCF. The blue, green, and red lines represent the calculated SNR dominated by the detector noise (SNR_{det}), the shot noise determined by the number of photons before the optical amplification with an EDFA ($SNR_{shot\text{-}UC}$), and the shot noise determined by the number of photons captured by a photodetector (SNR_{shot}), respectively. The orange line represents the upper limit of the average detection power determined by the detector's linearity. The SNR linearly increases against the average detection power due to the detector noise, while it is saturated around ten due to the shot noise determined by the number of photons before the EDFA and the amplifier noise.

References

1. Agrawal, G. P. *Nonlinear fiber optics*. (Academic Press, 2019)
2. Davis, S. P., Abrams, M. C. & Brault, J. W. *Fourier Transform Spectrometry.* (Academic Press, 2001).
